# Real-time PCR TaqMan assay for rapid screening of bloodstream infection

**DOI:** 10.1186/1476-0711-13-3

**Published:** 2014-01-07

**Authors:** Hye-young Wang, Sunghyun Kim, Hyunjung Kim, Jungho Kim, Yeun Kim, Soon-Deok Park, Hyunwoo Jin, Yeonim Choi, Young Uh, Hyeyoung Lee

**Affiliations:** 1 M&D, Inc., Wonju Eco Environmental Technology Center, Wonju, Gangwon 200-722, Republic of Korea; 2 Department of Biomedical Laboratory Science, College of Health Sciences, Yonsei University, Wonju, Gangwon 220-710, Republic of Korea; 3 Institute for Life Science and Biotechnology, Yonsei University, Seoul 120-749, Republic of Korea; 4 Department of Laboratory Medicine, Yonsei University Wonju College of Medicine, Wonju, Gangwon 220-701, Republic of Korea; 5 Department of Clinical Laboratory Science, College of Health Sciences, Catholic University of Pusan, Busan 609-757, Republic of Korea; 6 Department of Biomedical Laboratory Science, Songho College, Hoengseong 225-704, Republic of Korea

**Keywords:** Real-time polymerase chain reaction, Blood culture, Gram-positive bacteria, Gram-negative bacteria, *Candida*

## Abstract

**Background:**

Sepsis is one of the main causes of mortality and morbidity. The rapid detection of pathogens in blood of septic patients is essential for adequate antimicrobial therapy and better prognosis. This study aimed to accelerate the detection and discrimination of Gram-positive (GP) and Gram-negative (GN) bacteria and *Candida* species in blood culture samples by molecular methods.

**Methods:**

The Real-GP®, -GN®, and -CAN® real-time PCR kit (M&D, Wonju, Republic of Korea) assays use the TaqMan probes for detecting pan-GP, pan-GN, and pan-*Candida* species, respectively. The diagnostic performances of the real-time PCR kits were evaluated with 115 clinical isolates, 256 positive and 200 negative blood culture bottle samples, and the data were compared to results obtained from conventional blood culture.

**Results:**

Eighty-seven reference strains and 115 clinical isolates were correctly identified with specific probes corresponding to GP-bacteria, GN-bacteria and *Candida*, respectively. The overall sensitivity and specificity of the real-time PCR kit with blood culture samples were 99.6% and 89.5%, respectively.

**Conclusions:**

The Real-GP®, -GN®, and -CAN® real-time PCR kits could be useful tools for the rapid and accurate screening of bloodstream infections (BSIs).

## Background

The incidence of sepsis in the United States is 240.4 cases per 100,000 people with an 8.7% annual increase during the last several years [1]. The mortality rate of sepsis ranges between 21% and 55%, and it has been unchanged during the last decade [2,3].

Gram-positive (GP) bacteria are the most frequent causative agents of bloodstream infections (BSIs) (30-50% of all cases), followed by Gram-negative (GN) bacteria in 25-30% and fungal infections representing 1-3% of all sepsis cases [4]. Although rapid and accurate diagnosis of sepsis plays an important role in the reduction of mortality caused by sepsis, at least in 30% of sepsis cases, the causative pathogen could not be detected [4]. Currently, blood culture is the standard method for the diagnosis of bacteremia. However, the final results from identification/antimicrobial susceptibility tests in the continuous monitoring blood culture system (CMBCS) require at least 48 to 72 hrs [5]. Moreover, the CMBCS may cause false-negative results when fastidious or slowly growing organisms are the causative pathogens or when blood specimens are collected after antimicrobial therapy has been started [6,7]. In order to reduce the turnaround time for blood culture, a number of molecular methods for rapid identification of pathogens in positive blood culture samples have been tried, including DNA microarrays [8], RNA-based fluorescence in situ hybridization probes [9], and PCR-based assays like real-time PCR [10,11]. Among these methods, PCR-based assays have been reported to provide an early and accurate diagnosis of bacteremia and candidemia [12]. Sequence analysis of the PCR product is time-consuming, but has improved the rate of microbial detection. The sequence of the 16S rRNA gene has been used to diagnose and identify bacterial infection in clinical practice [13,14]. Some PCR-based assays could identify specific bacterial pathogens [10,11], while broad range bacterial PCR can detect almost any bacterial species [15,16]. Broad-range bacterial PCR has a great advantage in that it is able to detect microorganisms that are found less frequently, or can even identify unknown causative agents of bacterial origin.

However, conventional PCR is inconvenient for use in routine rapid screening due to the time required for sample handling and the risk of contamination in post-PCR analysis. Thus, it is necessary to develop a reliable broad-range detection system for bacterial and fungal genomic DNA from clinical samples that is fast, easy to use and covers a wide range of clinically relevant microbes. Additionally, the simultaneous quantification and differentiation of a Gram stain in clinical blood samples with a broad-range real-time PCR assay is rarely described.

In this study, we used a GP-bacteria, GN-bacteria and *Candida*-specific TaqMan probe-based real-time PCR system, which targets the bacterial 16S rRNA gene and fungal 18S rRNA gene, allowing simultaneous detection and discrimination of clinically-relevant GP-bacteria, GN-bacteria and *Candida* species in a total of 87 reference strains, 115 clinical isolates and 456 blood culture bottle samples from BSI-suspected patients.

## Materials and methods

### Bacterial and fungal strains

In this study, to determine the specificity of the Real-GP®, -GN®, and -CAN® real-time PCR kit (M&D, Wonju, Republic of Korea), a total of 62 bacterial, 25 fungal reference strains (Table 1), and 115 clinical isolates from various specimen types were used (Table 2). To evaluate the performance of the Real-GP®, -GN®, and -CAN® real-time PCR assay with blood culture bottle samples, a total of 456 samples including 256 positive and 200 negative blood culture samples were collected. All bacterial strains and clinical specimens were collected from December of 2011 to January of 2013 at Yonsei University Wonju Severance Christian Hospital, Wonju, Republic of Korea. All bacterial strains except *Mycobacterium* spp. were grown on sheep blood agar and MacConkey agar (BD Diagnostic System, Spark, MD, USA) at 37°C overnight and identified by the microplate method [17], the MicroScan® system (Siemens Healthcare Diagnostics, Sacramento, CA, USA), and the Vitek 2 system (bioMérieux, Durham, NC, USA). All mycobacterial reference strains were grown on Lowenstein-Jensen media (Union Lab, Seoul, Republic of Korea) at 37°C under 5% CO2 for 7 days to 8 weeks at Department of Microbiology, College of Medicine, Yonsei University, Seoul, Republic of Korea. All fungal reference strains were grown on Saboraud dextrose agar (BD Diagnostic System, Spark, MD, USA) at 25°C for several days at Korea Culture Collection Medical Fungi (KCMF), Konyang University, Daejeon, Republic of Korea.

**Table 1:** The specificity of Real-GP®, -GN®, and -CAN® real-time PCR assays for 62 bacterial and 25 fungal reference strains

**Genus**	**Species**	**Standard strains**	**Real-time PCR TaqMan assay (C**_**T **_**value)**		
			**Real-GP®**	**Real-GN®**	**Real-CAN®**
**Gram-positive bacteria**					
*Staphylococcus*	*S. aureus*	29213	26.59	UD	UD
	*S. aureus*	25923	28.42	UD	UD
	*S. xylosus*	29971	20.81	UD	UD
*Enterococcus*	*E. hirae*	9790	26.31	UD	UD
	*E. raffinosus*	49427	28.09	UD	UD
	*E. sulfureus*	49903	28.40	UD	UD
	*E. durans*	19432	23.48	UD	UD
	*E. casseliflavus*	700327	25.98	UD	UD
	*E. faecium*	19434	26.72	UD	UD
	*E. faecalis*	29212	25.33	UD	UD
	*E. mundtii*	43186	29.90	UD	UD
	*E. cecorum*	43198	21.68	UD	UD
	*E. flavescens*	49997	22.21	UD	UD
	*E. gallinarum*	49573	23.88	UD	UD
	*E. faecalis*	51299	24.10	UD	UD
	*E. solitarius*	49428	30.44	UD	UD
	*E. faecium*	35667	24.66	UD	UD
	*E. malodoratus*	43197	27.02	UD	UD
	*E. saccharolyticus*	43076	23.06	UD	UD
	*E. casseliflavus*	25788	26.28	UD	UD
*Streptococcus*	*S. pneumoniae*	49619	21.11	UD	UD
	*S. agalactiae*	13813	26.41	UD	UD
*Micrococcus*	*M. luteus*	49732	22.36	UD	UD
*Mycobacterium*	*M. avium*	25291	23.06	UD	UD
	*M. chelonae*	35749	21.34	UD	UD
	*M. gastri*	15754	23.90	UD	UD
	*M. kansasii*	12478	22.17	UD	UD
	*M. nonchromogenicum*	19530	18.31	UD	UD
	*M. phlei*	11758	25.09	UD	UD
	*M. smegmatis*	19420	24.40	UD	UD
	*M. triviale*	23292	22.66	UD	UD
	*M. aurum*	23366	24.83	UD	UD
	*M. farcinogen*	35753	20.00	UD	UD
	*M. gilvum*	43909	19.40	UD	UD
	*M. neoaurum*	25795	17.83	UD	UD
	*M. parafortuitum*	19686	19.06	UD	UD
	*M. peregrinum*	14467	18.57	UD	UD
	*M. septicum*	700731	23.47	UD	UD
	*M. abscessus*	19977	21.73	UD	UD
**Gram-negative bacteria**					
*Escherichia*	*E. coli*	25922	UD	20.46	UD
	*E. coli*	35218	UD	17.90	UD
*Enterobacter*	*E. aerogenes*	1304	UD	19.09	UD
*Citrobacter*	*C. freundii*	6750	UD	14.19	UD
*Shigella*	*S. boydii*	DML 399	UD	25.59	UD
	*S. dysenteriae*	DML 400	UD	18.38	UD
	*S. flexneri*	9199	UD	21.82	UD
*Serratia*	*S. liquifaciens*	27952	UD	24.96	UD
*Salmonella*	*S. typhi*	19430	UD	16.54	UD
	*S. enteritidis*	13076	UD	20.15	UD
	*S. paratyphi*	11511	UD	19.61	UD
	*S. typhimurium*	13311	UD	16.19	UD
	*S. newport*	6962	UD	17.22	UD
*Klebsiella*	*K. pneumoniae*	13883	UD	20.60	UD
	*K. oxytoca*	700324	UD	21.87	UD
*Proteus*	*P. alcalifaciens*	51902	UD	18.38	UD
	*P. vulgaris*	49132	UD	17.13	UD
	*P. mirabilis*	49132	UD	16.34	UD
*Pseudomonas*	*P. cepacia*	25608	UD	19.66	UD
	*P. aeruginosa*	27853	UD	16.57	UD
*Haemophilus*	*H. influenzae*	49247	UD	18.61	UD
*Leclercia*	*L. adecarboxylata*	23216	UD	15.70	UD
*Bordetella*	*B. bronchiseptica*	10580	UD	19.44	UD
**Fungi**					
*Penicillium*	*P. camemberti*	58608	UD	UD	UD
	*P. paneum*	KACC 44823	UD	UD	UD
*Aspergillus*	*A. oryzae* var *oryzae*	KACC 44847	UD	UD	UD
	*A. oryzae* var. *effusus*	1010	UD	UD	UD
	*A. clavatus*	66443	UD	UD	UD
	*A. sydowii*	KACC 41869	UD	UD	UD
	*A. fumigatus*	KCMF 10773	UD	UD	UD
	*A. flavus*	KCMF 10777	UD	UD	UD
	*A. tamari*	20054	UD	UD	UD
*Fusarium*	*F. acuminatum*	10466	UD	UD	UD
*Aureobasidium*	*A. pullulans*	KACC 41291	UD	UD	UD
*Bipolaris*	*B. sorokiniana*	KACC 44841	UD	UD	UD
*Cryptococcus*	*C. neoformans*	KCMF 20047	UD	UD	UD
*Kodamea*	*K. ohmeri*	KCMF 20430	UD	UD	UD
*Saccaromyces*	*S. cerevisiae*	KCMF 50427	UD	UD	UD
*Trichophyton*	*T. rubrum*	KCMF 10444	UD	UD	UD
	*T. mentagrophytes*	KCMF 10515	UD	UD	UD
*Microsporum*	*M. canis*	KCMF 10531	UD	UD	UD
*Epidermophyton*	*E. floccosum*	52063	UD	UD	UD
*Malassezia*	*M. furfur*	KCMF 20409	UD	UD	UD
*Candida*	*C. albicans*	36802	UD	UD	26.42
	*C. tropicalis*	14506	UD	UD	25.98
	*C. glabrata*	38326	UD	UD	17.09
	*C. parapsilosis*	7330	UD	UD	24.27
	*C. krusei*	20298	UD	UD	19.67

*Abbreviations:ATCC* American type culture collection*, DML* Diagnostic Microbiology Laboratory*,* Biomedical laboratory science, Yonsei University*; KACC* Korean Agricultural Culture Collection*; KCMF* Korea Culture Collection Medical Fungi; *UD* Undetermined.

**Table 2:** **Real-GP®, -GN®, and -CAN® real-time PCR assay results for discriminating the Gram-positive and -negative bacteria and *Candida *** **species in 115 clinical isolates**

**Culture identification**	**No. of samples (n)**	**Real-time PCR TaqMan assay**		
		**GP/GN or *Candida***	**Ranged C**_**T **_**value**	**Mean C**_**T **_**value**
*Staphylococcus aureus*	12	GP	22.44-26.65	24.47
*Staphylococcus* spp. (CoNS)	8	GP	19.46-28.71	21.5
*Streptococcus* spp.	5	GP	17.35-30.46	24.25
*Enterococcus faecalis*	4	GP	25.2-27.3	26.37
*Enterococcus faecium*	10	GP	21.3-31.6	26.58
*Enterococcus mundtii*	1	GP	27.85	27.85
*Corynebacterium* spp.	1	GP	24.51	24.51
*Escherichia coli*	16	GN	12.68-30.65	23.26
*Klebsiella pneumoniae*	13	GN	15.48-26.08	20.73
*Pseudomonas aeruginosa*	13	GN	15.23-19.96	18.11
*Acinetobacter baumannii*	11	GN	18.09-24.65	21.04
*Enterobacter asburiae*	2	GN	15.79	15.79
*Enterocobacter cloacae*	1	GN	15.41	15.41
*Moraxella catarrhalis*	1	GN	33.54	33.54
*Serratia marcescens*	1	GN	21.64	21.64
*Providencia rettgeri*	1	GN	24.3	24.30
*Morganella morganii*	1	GN	20.6	20.60
*Proteus mirabilis*	1	GN	24.88	24.88
*Aeromonas* spp.	1	GN	25.97	25.97
*Citrobacter fruendii*	2	GN	17.11-18.01	17.56
*Candida albicans*	5	CAN	17.61-29.56	23.9
*Candida parapsilosis*	3	CAN	24.73-30.95	27.58
*Candida tropicalis*	1	CAN	26.62	26.62
*Candida glabrata*	1	CAN	17.68	17.68
**Total**	115			

### Blood culture and collection of blood culture bottle samples

Three or two pairs of culture bottles for aerobes or anaerobes were incubated in the BacT/Alert 3D (bioMérieux) and BACTEC® 9240 system (Becton Dickinson Diagnostic System, Spark, MD, USA) or BACTEC FX (Becton Dickinson) blood culture systems for 5 days after inoculating blood drawn from the patient at the bedside. If no bacterial growth was detected within 5 days, the blood culture was considered negative. When bacterial growth was noted, the culture sample was inoculated into blood and MacConkey agar plates (BD Diagnostic Systems, Sparks, MD, USA), and then cultured overnight at 37°C in a 5% CO_2_ incubator. Isolates were identified based on the colony morphology, Gram stain, biochemical tests, and commercial identification kits. MicroScan® (Siemens Healthcare Diagnostics, Sacramento, CA, USA) overnight Pos BP Combo 28, MICroSTREP Plus, overnight Neg Combo 53, and Neg Combo 54 panels were used for the identification of GP, streptococci, and GN bacteria. For identification of *Candida* spp., a VITEK-2 (bioMérieux, Marcy l’Etoile, France) YST ID CARD was used.

### DNA preparation

To prepare DNA templates for the real-time PCR TaqMan assay, one colony of each strain and clinical isolate was suspended in 100 μL of DNA extraction solution (M&D, Wonju, Republic of Korea). The suspended bacterial solution was boiled for 10 min. After centrifugation at 13,000 g for 10 min, the supernatant was used for DNA templates.

For preparation of DNA template from the blood culture bottle samples, 0.5 mL of blood suspension were taken directly from the blood culture bottle, and 1 mL of phosphate-buffered saline (pH 8.0) was added and centrifuged at 13,000 g for 1 min. The supernatant was removed, and the pellet was resuspended in 1 mL of ACK solution (0.15 M of NH_4_Cl, 1 mM of KHCO_3_, and 0.1 mM of Na_2_EDTA), and centrifuged at 13,000 g for 1 min. This washing step was repeated twice, and the pellet was resuspended in DNA extraction solution as described above for the clinical isolates.

### Real-time PCR TaqMan assay

The real-time PCR TaqMan assay was carried out with the Real-GP®, -GN® and -CAN® real-time PCR assay kits (M&D), and a CFX-96 real-time PCR system (Bio-rad, Hercules, CA, USA) and an ABI 7500 FAST instrument (Applied Biosystem, Foster City, CA, USA) were used for the thermo-cycling and fluorescence detection. These real-time PCR assay kits are only able to determine GP bacteria, GN bacteria, and *Candida*, respectively for rapid screening of BSIs however they do not allow species or genus identification and antimicrobial susceptibility. The real-time PCR amplification was performed in a total volume of 25 μL that contained 12.5 μL of 2 × Thunderbird probe qPCR mix (Toyobo, Osaka, Japan), 5 μL of primer and TaqMan probe mixture, 5 μL of template DNA, and ddH_2_O was added to give a final volume of 25 μL for each sample.

Positive and negative controls were included throughout the procedure. No-template controls with ddH_2_O instead of template DNA were incorporated in each run under the following conditions: 95°C for 3 min and 40 cycles of 95°C for 20 s and 60°C for 40 s in single real-time PCR. The bacterial load was quantified by determining the cycle threshold (C_T_), the number of PCR cycles required for the fluorescence to exceed a value significantly higher than the background fluorescence.

## Results

### Sensitivity and specificity of the Real-GP®, -GN®, and -CAN® real-time PCR TaqMan assay with reference bacterial and fungal strains

**Figure 1:**
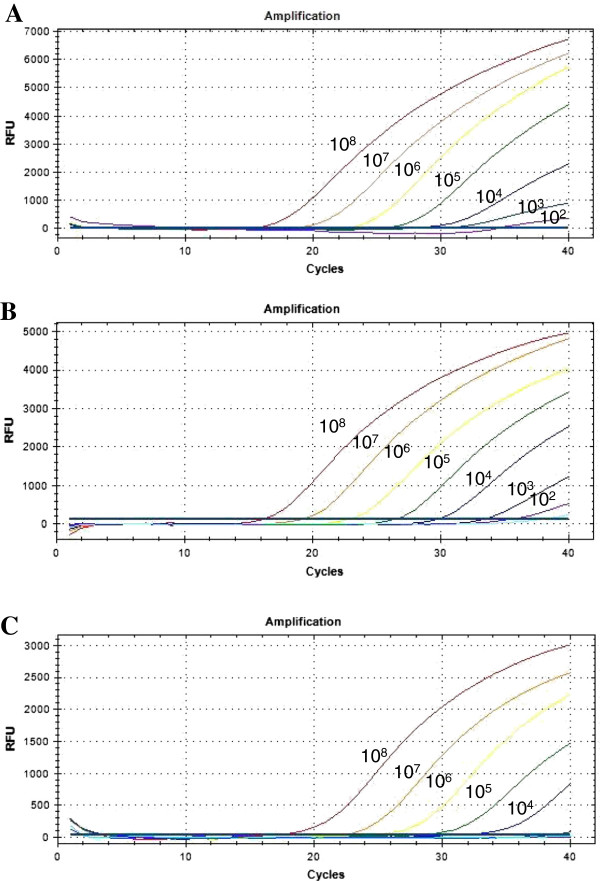
**Detection limits of the three target probes from 10-fold serial diluted spiked samples.** Serially diluted DNA amounts ranging from 10^8^ to 10° CFU/mL were used to determine the detection limit of the multiplex real-time PCR assay. **(A)** amplification curve of GP-bacteria probe using *Staphylococcus aureus*, **(B)** amplification curve of GN-bacteria probe using *Escherichia coli*, **(C)** amplification curve of *Candida* probe using *Candida glabrata*. The overall detection limit of this assay for GP-*,* GN-bacteria, and *Candida* probe was approximately 10^3^ to 10^4^ CFU/mL.

The detection limit of the real-time PCR TaqMan assay for GP-, GN-bacteria*,* and *Candida* was 10^3^ CFU/mL, 10^3^ CFU/mL, and 10^4^ CFU/mL, respectively. The C_T_ values for GP-, GN-bacteria*,* and *Candida* with each cell concentrate (10^8^ - 10^2^ CFU/mL) ranged from 16.17 to 32.46, 15.06 to 29.03, and 17.68 to 32.47, respectively (Figure 1).

All DNA extractions of bacterial and fungal reference strains showed the positive fluorescence signals with real-time PCR TaqMan assay. The C_T_ values for GP-, GN-bacteria and *Candida* real-time PCR assays ranged from 17.83 to 30.44, 14.19 to 25.59, and 17.09 to 26.42, respectively (Table 1).

### Results of Real-GP®, -GN®, and -CAN® real-time PCR TaqMan assay with clinical isolates

The results between subculture and real-time PCR assay were completely concordant (100%) in 115 clinical isolates. Forty-one GP clinical isolates, which included 12 *Staphylococcus aureus*, 10 *Enterococcus faecium*, 8 coagulase-negative staphylococci (CoNS), 5 *Streptococcus* spp., 4 *E. faecalis*, 1 *Enterococcus mundtii*, and 1 *Corynebacterium* spp., were positive by Real-GP**®** assay and sixty-four GN clinical isolates, which included 16 *Escherichia. coli*, 13 *Klebsiella pneumoniae*, 13 *Pseudomonas aeruginosa*, 11 *Acinetobacter baumannii*, two *Citrobacter fruendii,* two *Enterobacter asburiae,* one *Enterobacter coloacae*, one *Aeromonas* spp., one *Moraxella catarrhalis*, one *Morganella morganii*, one *Proteus mirabilis*, one *Providencia rettgeri*, and one *Serratia marcescens,* were positive by Real-GN**®** assay*.* Ten *Candida* spp. clinical isolates, which included five *C. albicans,* three *C. parapsilosis,* one *C. tropicalis* and one *C. glabrata,* were positive by Real-CAN**®** assay (Table 2). The C_T_ values of clinical isolates of GP, GN, and *Candida* species ranged from 17.35 to 31.60, 12.68 to 33.54, and 17.61 to 30.95, respectively.

### Results of Real-GP®, -GN®, and -CAN® real-time PCR TaqMan assay with positive and negative blood culture bottle samples

Of 256 positive blood culture bottles, GP-bacteria, GN-bacteria, and *Candida* were detected in 175, 70, and four bottles, respectively. Two kinds of microorganisms were detected from six bottles. Among 175 GP-bacteria-positive blood cultures, *Staphylococcus epidermidis* was the most prevalent at 26.7% (n = 47), followed by *S. aureus* (n = 24), *Staphylococcus hominis* (n = 17), GP rods (n = 16), and *Staphylococcus capitis* (n = 14). One *S. hominis* showed a negative result with real-time PCR. The C_T_ values of 175 GP-bacteria ranged from 11.52 to 34.39. Seventy GN-bacteria from blood cultures included *Escherichia coli* (50%, n = 35), *Klebsiella pneumoniae* (n = 13), *Acinetobacter baumannii* (n = 5), and *Pseudomonas aeruginosa* (n = 3). The C_T_ values of the 70 GN-bacteria ranged from 6.56 to 24.08. A total of four *Candida*-positive samples included two *C. albicans*, one *C. parapsilosis* and one *C. tropicalis* (Table 3). The C_T_ values of the four *Candida* species ranged from 22.81 to 31.98.

**Table 3:** Comparison of the results of Real-GP®, -GN®, and -CAN® real-time PCR assay and BACTEC 9240 for detection of bloodstream infection in positive and negative blood culture bottle samples

**Blood culture result**	**Real-time PCR TaqMan assay (n)**		**GP**	**GN**	**CAN**
	**Positive**	**Negative**	**(C**_**T **_**range)**	**(C**_**T **_**range)**	**(C**_**T **_**range)**
**Blood culture positive (n = 256)**	255	1			
**Gram-positive bacteria (n = 176)**	175	1			
*Staphylococccus epidermidis* (47)	47	0	13.08-33.99	UD	UD
*S. aureus* (24)	24	0	13.24-34.19	UD	UD
*S. hominis *(17)	16	1	13.69-30.00	UD	UD
*S. capitis *(14)	14	0	13.60-25.10	UD	UD
*S. haemolyticus* (8)	8	0	14.68-33.30	UD	UD
*S. warneri* (1)	1	0	18.50	UD	UD
*S. saprophyticus* (1)	1	0	16.57	UD	UD
*S. xylosus* (1)	1	0	21.23	UD	UD
*S. chleiferi* (1)	1	0	20.44	UD	UD
*Streptococcus salivarius* (5)	5	0	13.55-23.58	UD	UD
*S. mitis* (4)	4	0	11.52-23.12	UD	UD
*S. pneumoniae* (4)	4	0	16.37-17.77	UD	UD
*S. agalactiae* (2)	2	0	21.52-25.95	UD	UD
*S. pyogenes* (1)	1	0	22.10	UD	UD
*S. dysgalactiae* (1)	1	0	15.81	UD	UD
*S. parasangus* (1)	1	0	12.96	UD	UD
*Streptococcus* spp. (2)	2	0	17.87-24.89	UD	UD
*Enterococcus faecium* (8)	8	0	26.43-27.54	UD	UD
*E. faecalis* (1)	1	0	14.50	UD	UD
*Micrococcus* spp. (5)	5	0	20.96-31.33	UD	UD
*Propionibacterium acnes* (3)	3	0	23.77-26.86	UD	UD
*Peptostreptococcus asaccharolyticus* (1)	1	0	26.72	UD	UD
*Peptostreptococcus micros* (1)	1	0	26.00	UD	UD
*Corynebacterium* spp*.* (6)	6	0	26.47	UD	UD
Gram positive rods (16)	16	0	12.43-34.39	UD	UD
**Gram-negative bacteria (n = 70)**	70	0			
*Escherichia coli* (35)	35	0	UD	9.86-21.89	UD
*Klebsiella pneumoniae* (13)	13	0	UD	12.84-13.20	UD
*Acinetobacter baumannii* (5)	5	0	UD	10.53-11.89	UD
*A. woffii* (1)	1	0	UD	12.22-15.11	UD
*Enterobacter* spp. (2)	2	0	UD	6.56	UD
*Pseudomonas aeruginosa* (3)	3	0	UD	12.08	UD
*Salmonella* group D (1)	1	0	UD	13.62-21.48	UD
*Proteus mirabilis* (1)	1	0	UD	14.38	UD
*Aeromonas* spp. (2)	2	0	UD	20.10	UD
*Morganella morganii* (1)	1	0	UD	10.39	UD
*Haemophillus influenzae* (1)	1	0	UD	20.68	UD
*Chryseobacterium indologenes* (1)	1	0	UD	14.40	UD
*Sphingomonas paucimobilis* (1)	1	0	UD	24.08	UD
*Serratia marcescens* (1)	1	0	UD	9.48	UD
*Citrobacter freundii* (1)	1	0	UD	14.24	UD
***Candida *** **species (n = 4)**	4	0			
*Candida albicans* (2)	2	0	UD	UD	26.98-31.52
*C. parapsilosis* (1)	1	0	UD	UD	31.98
*C. tropicalis* (1)	1	0	UD	UD	22.81
*Polymicrobial infection (n = 6)	6	0			
*Streptococcus agalactiae, Citrobacter koseri* (1)	1	-	14.61	10.61	UD
*Enterococcus faecium, Candida albicans* (1)	1	-	17.18	UD	29.51
*Enterococcus faecalis, Proteus mirabilis* (1)	1	-	UD	12.14	UD
*Escherichia coli, Enterococcus gallinarym* (1)	1	-	UD	13.25	UD
*Klebsiella pneumonia, Enterococcus casseliflavus* (1)	1	-	UD	13.31	UD
*Klebsiella pneumonia, Enterobacter cloacae* (1)	1	-	UD	14.42	UD
**Blood culture negative (n = 200)**	21	179			
Gram-positive bacteria	7	193	17.54-27.43	-	-
Gram-negative bacteria	14	186	-	20.48-31.26	-
*Candida* species	0	200	-	-	-

*GP-, GN-bacteria or fungi mixed infection.

Among six cases of polymicrobial bacteremia, three were concordant between the standard identification method and real-time PCR assay, but the other three cases showed positive results for only a single organism by real-time PCR assay (Table 3).

Of 200 negative blood culture bottle samples, 21 samples (10.5%), including seven GP- and 14 GN-bacteria, had positive results with the real-time PCR assay (Table 3). The C_T_ values of 7 GP-bacteria and 14 GN-bacteria ranged from 17.54 to 23.28, and 17.79 to 31.26, respectively.

The sensitivity of the real-time PCR kit for GP, GN, *Candida* and polymicrobial isolates was 99.4%, 100%, 100% and 100%, respectively, and the specificity of the real-time PCR kit for GP, GN and *Candida* was 96.5%, 93.0% and 100%, respectively. Therefore, the overall sensitivity and specificity of the real-time PCR kit compared with blood culture method were 99.6% and 89.5%, respectively.

## Discussion

Blood culture is the current gold standard method for detecting BSI microbial pathogens; although it allows microbes to be identified and their susceptibility profiles to be tested, the method has several limitations. Lack of rapidity is a major problem because detection of bacterial and fungal growth requires approximately 12 to 48 hr, and it can take more time in the case of fastidious bacterial or invasive fungal infection [18,19].

Currently, novel diagnostic technique such as MALDI-TOF MS was developed and evaluated with positive blood culture bottle samples [20,21]. It could provide species or genus identification more rapidly for detecting BSIs than current molecular assays however it cannot replace to routine laboratory workflow in current clinical setting yet because of the cost, facility, and examiner. Current DNA-based Gram identification methods include Gram stain-specific PCR [22], nested PCR [23], and PCR probe hybridization [24-26], but all of these methods contain at least two sequential steps, and therefore they require a longer turnaround time to get a final result. For instance, the conventional PCR assays incorporate a pair of oligonucleotide primers to amplify a specific target gene that is then detected using agarose gel electrophoresis combined with an intercalating dye (e.g., ethidium bromide, EtBr) and UV light. The real-time PCR TaqMan assay is a promising tool for detecting bacterial genomic DNA from biological fluids (direct clinical specimens) such as blood, urine and sputum. Fluorescence hybridization probes result in fast detection of small amounts of bacterial genomic DNA and correct Gram classification [27]. It is not only applicable from one sample to a number of samples at once but also it can be commonly used for diagnostic purpose in current clinical laboratories cost effectively.

In this study, Real-GP®, -GN® and -CAN® real-time PCR TaqMan assays, which target the bacterial 16S rRNA and fungal 18S rRNA, were evaluated with reference bacterial and fungal strains, clinical isolates, and direct blood culture bottle samples. The results showed that the real-time PCR TaqMan assay was rapid; it usually took no more than 4 hr to complete the whole experiment, which included only 1 hr of sample preparation and 1.5 hr for DNA amplification, because thermal cycling is much faster and amplicon detection is performed in real time. It allowed for the rapid quantification and Gram classification of bacteria and fungi without the post-PCR process. Furthermore, it was very specific because the results were completely accurate when compared with the standard blood culture method.

In previous reports from other study groups, CoNS were reported to be the major causative microorganisms in sepsis [28,29]. In this study, *S. epidermidis* was identified as the most common in positive blood culture bottle samples, with a total of 47 cases, which is identical to the results from other studies. Therefore, future study of the role and the effect of S. *epidermidis* infection in the bloodstream might be essential in the Korean population. All GN-bacteria and *Candida* samples were positive and all GP-bacteria samples, except for one sample, were positive based on real-time PCR assay in clinical isolates and positive blood culture samples. Therefore, the sensitivity was sufficient for performance with positive blood culture bottle samples. Also, candidiasis is a yeast infection caused by different *Candida* species and can cause opportunistic infections of the skin and mucosa as well invasive infections. Candidiasis accounts for up to 10% of bloodstream infections and is associated with an exceptionally high mortality rate [30]. Invasive fungal infections are increasingly recognized as a primary cause of morbidity and mortality especially in immunocompromised patients. To reduce mortality in patients with invasive candidiasis, early diagnosis and rapid initiation of antifungal therapy is very important for the survival of patients. However, our research has shown that the test was just 4/256 (1.6%) in *Candida* species therefore it is necessary to be carried out more tests with larger number of samples. The positivity of the Real-GP®, -GN®, -CAN® real-time PCR assay (21/200; 10.5%) was significantly higher than that of blood culture (0/200; 0%). When blood culture was used as a standard control, the sensitivity of real-time PCR was 100% and the specificity was 89.5% (Table 4). The gold standard for diagnosing sepsis is now still blood culture, even though, in many cases, blood cultures are negative in the face of strong clinical indicators of sepsis [31]. Therefore, to effectively evaluate the real-time PCR kit for rapid screening of BSI, further evaluation is required with direct blood samples or a larger number of negative blood culture bottle samples from patients who are suspected to have sepsis.

**Table 4:** The sensitivity and specificity of the Real-GP®, -GN®, and -CAN® real-time PCR assay with positive and negative blood culture bottle samples

**Blood culture**	**Real-time PCR**		**Sensitivity**	**Specificity**
	**TaqMan assay (n)**			
	**Positive**	**Negative**		
**Blood culture positive (n = 256)**	255	1	99.6%	**-**
Gram-positive bacteria (176)	175	1	99.4%	-
Gram-negative bacteria (70)	70	0	100%	-
*Candida* species (4)	4	0	100%	-
Multiple infection (6)	6	0	100%	-
**Blood culture negative (n = 200)**	21	179	-	89.5%
Gram-positive bacteria	7	193	-	96.5%
Gram-negative bacteria	14	186	-	93.0%
*Candida* species	0	200	-	100%

Polymicrobial culture data show that six samples were infected with two microorganisms each. Among these six cases, the results of three were consistent between conventional culture and real-time PCR assay (showed double signal), however, three samples showed just a single positive signal with real-time PCR, even though two microorganisms were identified by the culture method. According to the real-time PCR results, GN signals were positive and GP signals were undetermined in those three samples. The reason might be due to the fact that the GP-bacteria, *Enterococcus* spp., grow more slowly, and thus, the number of GP-bacteria is much fewer than that of GN bacteria (*E. coli* and *K. pneumoniae*) in blood culture bottle samples, and the fluorescence signal for GN could not be detected.

## Conclusions

The use of the molecular diagnostic assay, real-time PCR TaqMan assay, is more effective for rapid screening of BSI than microbiological diagnosis. In this study, this technique allowed the simultaneous detection, quantification, and Gram identification of bacterial and fungal organisms directly from blood culture bottle samples. Even culture, species or genus identification, and antimicrobial susceptibility testing could not be substituted by this real-time PCR TaqMan assay, it could not only differentiate bacterial and fungal from viral and other pathogens, but it could also classify Gram staining with a much shorter turnaround time than the gold standard culture method.

The Real-GP®, -GN®, and -CAN® real-time PCR assays may provide essential information to accelerate therapeutic decisions for earlier and adequate antibiotic treatment in the acute phase of sepsis.

## Competing interests

The authors declare that they have no competing interests.

## Authors’ contributions

HYW and SK carried out data organization and analysis and contributed to writing and to the interpretation of the results. HK, JK, YK, SDP, HJ, and YC carried out the evaluation of experiments and collected all clinical samples and data. HYL and YU contributed to the design of the study and assisted in the drafting of the manuscript. All authors have read and approved the manuscript.
